# Low‐level lysosomal membrane permeabilization for limited release and sublethal functions of cathepsin proteases in the cytosol and nucleus

**DOI:** 10.1002/2211-5463.13385

**Published:** 2022-03-09

**Authors:** Thomas Reinheckel, Martina Tholen

**Affiliations:** ^1^ Institute of Molecular Medicine and Cell Research Faculty of Medicine Albert‐Ludwigs‐University Freiburg Germany; ^2^ German Cancer Consortium (DKTK), Partner Site Freiburg Germany; ^3^ German Cancer Research Center (DKFZ) Heidelberg Germany; ^4^ Center for Biological Signaling Studies BIOSS Albert‐Ludwigs‐University Freiburg Germany

**Keywords:** cathepsin, cell cycle, cell death, lysosome, protease

## Abstract

For a long time, lysosomes were purely seen as organelles in charge of garbage disposal within the cell. They destroy any cargo delivered into their lumen with a plethora of highly potent hydrolytic enzymes, including various proteases. In case of damage to their limiting membranes, the lysosomes release their soluble content with detrimental outcomes for the cell. In recent years, however, this view of the lysosome changed towards acknowledging it as a platform for integration of manifold intracellular and extracellular signals. Even impaired lysosomal membrane integrity is no longer considered to be a one‐way street to cell death. Increasing evidence suggests that lysosomal enzymes, mainly cathepsin proteases, can be released in a spatially and temporarily restricted manner that is compatible with cellular survival. This way, cathepsins can act in the cytosol and the nucleus, where they affect important cellular processes such as cell division. Here, we review this evidence and discuss the routes and molecular mechanisms by which the cathepsins may reach their unusual destination.

## Graphical abstract

Frequent reports describe the occurrence of lysosomal proteases in the cytosol or nucleus of cells, where they support processes like cell division, epigenetic regulation, or cytoskeletal rearrangement, but how do lysosomal enzymes escape their normal biogenesis and regular trafficking into the acidic cell compartment? In this review, we discuss the biogenesis routes and functions of nucleo‐cytosolic lysosomal proteases.

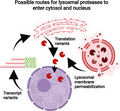

**Abbreviations**
4‐HNE: 4‐hydroxynonenal
5′UTR: 5′untranslated region
CDP/Cux: CCAAT‐displacement protein/cut homeobox
CTSB: cathepsin B
CTSC: cathepsin C
CTSF: cathepsin F
CTSK: cathepsin K
CTSL: cathepsin L
CTSO: cathepsin O
CTSS: cathepsin S
CTSV: cathepsin V
CTSW: cathepsin W
CTSZ: cathepsin Z
ER: endoplasmic reticulum
HSP: heat shock protein
IRES: internal ribosomal entry site
LMP: lysosomal membrane permeabilization
NCBI: National Center for Biotechnology Information
NLRP3: NLR‐family‐pyrin‐domain‐containing‐3
PI3K/mTORC1: phosphatidylinositol‐3‐kinase/mammalian target of rapamycin complex 1
TRPS1: tricho‐rhino‐phalangeal‐syndrome 1

## Introduction

**Fig. 1:**
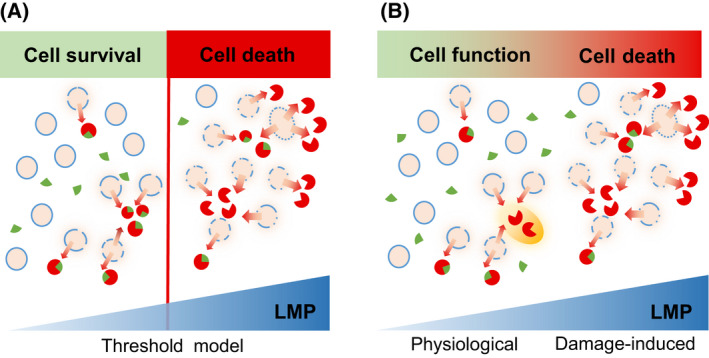
Models for cellular consequences of lysosomal membrane permeabilization (LMP). (A) In the classical ‘threshold model’, proteases leaking out of the lysosome are inhibited (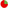) by cytosolic factors, e.g., the cystatins (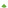) scavenging extralysosomal cysteine cathepsins. Lysosomal leakage exceeding the cellular scavenging capacity results in active proteases (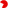), which leads inevitably to cell death. (B) The ‘continuity LMP’ model allows for spatially and temporally restricted activity of cysteine cathepsins (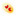), thereby promoting the physiological molecular fine‐tuning of important cellular processes, while extensive lysosomal damage results in cell death. In this ‘continuity model’, not only the quantity of cathepsin release but also its timing and location is of uttermost importance.

The classical function of the endosomal‐lysosomal compartment is to provide the perfect conditions for more than 50 highly active hydrolases executing the degradation of material that is imported into this compartment [1]. Hence, compartmentalization into endolysosomal vesicles has been frequently interpreted as a safeguard against the destructive potential of these hydrolytic enzymes in general and of lysosomal proteases in particular [2]. In the classical model, ectopic hydrolytic activity after release of proteases from the lysosome by lysosomal membrane permeabilization (LMP) is countered by the unfavorable cytosolic pH and the presence of endogenous inhibitors (Fig. 1A) [3, 4]. Nonetheless, massive release of hydrolases by LMP overpowers these cytosolic defense measures and results in apoptotic, pyroptotic, or even necrotic types of cell death [5, 6, 7]. Cell death events in which lysosomal leakage and ectopic action of lysosomal enzymes are the primary causal event are defined as lysosome‐dependent cell death [8, 9].

Despite this prevalent dogma, there are frequent reports of lysosomal proteases occurring in the cytosol or nucleus of cells without the induction of cell death [10, 11, 12]. It is thought that lysosomal proteases at such locations support processes of cell division, epigenetic regulation, or cytoskeletal rearrangement. Although those observations are ample, many conceptual questions remain. How do lysosomal enzymes escape their normal biogenesis route and vesicular compartment? Is this accidental or regulated? If it is accidental—how can a highly regulated process like cell division be affected by lysosomal protease release? How about endogenous cytosolic protease inhibitors? In this review, we will critically address those questions, with a special focus particularly on the abundant and numerous cysteine‐type cathepsins as prototypic lysosomal proteases.

## Synthesis and trafficking of lysosomal proteases

Lysosomal proteases are a heterogeneous group of enzymes comprising serine proteases (cathepsin A, cathepsin G, and tripeptidyl‐peptidase 1), aspartic proteases (cathepsin D, cathepsin E, and napsin A), and cysteine proteases (legumain and cysteine‐type cathepsins). Cysteine cathepsins, comprising the largest group of lysosomal proteases, are a family of papain‐like proteases (clan CA, family C1) [13]. The gene annotation of the cathepsins is ‘CTS’ followed by a letter indicating the specific enzyme, e.g., cathepsin B is abbreviated as CTSB. They form the largest family of lysosomal proteases with eleven members in humans, i.e., CTSB, CTSC, CTSF, CTSH, CTSL (also known as CTSL1), CTSK, CTSO, CTSS, CTSV (also known as CTSL2), CTSW, and CTSZ (also known as CTSX), and until now 22 members in mice [14, 15]. This family of proteolytic enzymes is characterized by a wide substrate specificity, which enables these proteases to degrade most intracellular and extracellular proteins delivered to the lysosome by endocytosis and autophagy [16]. Cysteine cathepsins mainly act as endopeptidases, like CTSL, with a few exceptions that show exopeptidase activity, like CTSH, CTSB, and CTSZ [15, 17].

**Fig. 2:**
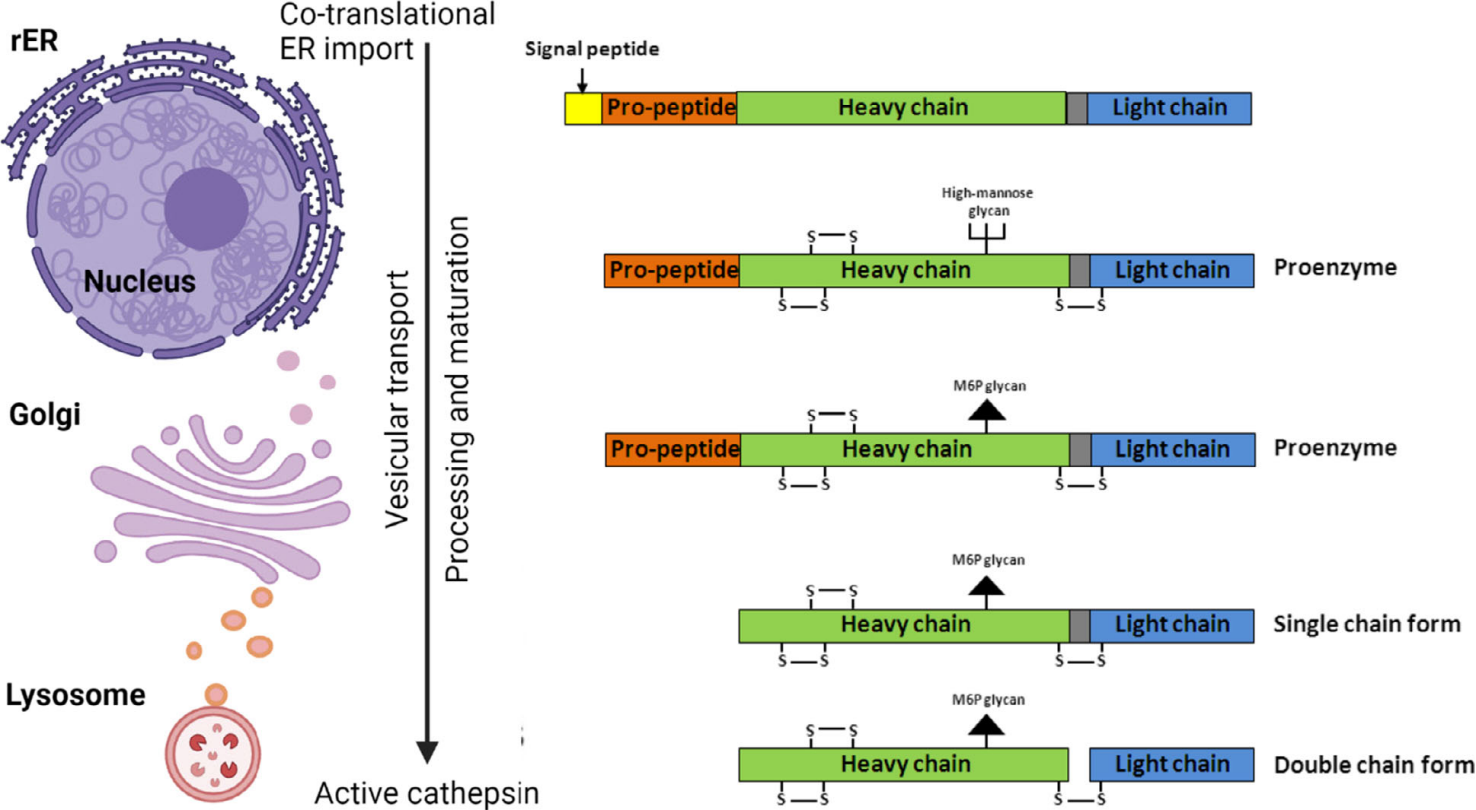
Trafficking and maturation of cathepsin L (CTSL). Trafficking and post‐translational modifications from the co‐translational import into the rough endoplasmatic reticulum (rER) to complete proteolytic activation in the lysosome. Note that removal of the signal peptide and propeptide as well as degeneration of double‐chain CTSL are specific proteolytic events needed for CTSL maturation. However, single‐chain and double‐chain CTSL are both proteolytically active. Figure created with Biorender.com.

The biosynthesis and intracellular trafficking of lysosomal proteases (depicted for CTSL in Fig. 2) is exemplary of how a cell regulates activity by restricting it to the correct cellular compartment [18]. Especially in case of proteases with wide substrate specificity, like cathepsins, this is a measure to avoid aberrant proteolytic activity. Cathepsins are synthesized at the rough ER as inactive preproenzymes [19]. They are targeted for entry into the ER by the ER‐import signal, where the import signal is cleaved off by a signal peptidase [20, 21]. After folding of the proforms, they are trafficked through the intermediate Golgi compartments to the trans‐Golgi network, where they undergo further post‐translational modifications like glycosylation and phosphorylation of mannose‐6 residues. In the trans‐Golgi network, binding of phosphorylated mannose‐6 residues to the mannose‐6 phosphate receptor mediates targeting of the procathepsins to the endosomal/lysosomal compartment [22, 23]. Maturation of the proenzymes takes place in the acidic endosomal/lysosomal compartment, where the proregion is cleaved off autocatalytically, or by legumain, the aspartic protease CTSD or other lysosomal proteases [15, 21]. The single‐chain form of the active enzyme is further processed into two chains that are connected via disulfide bonds [15, 24]. In the lysosome, cathepsins find optimal conditions to be active. CTSL, for example, is catalytically active at pH 3.0–6.5 with an optimum at pH 5.5 under reducing conditions [15, 25].

In the discussion of lysosomal protease functions outside these acidic organelles, it should be noted that recombinant lysosomal proteases are either completely inactive at cytosolic pH conditions (i.e., pH 7.2) or tend to change their cleavage preference, which is probably due to pH‐dependent structural distortion of the enzymes [26, 27]. This makes it difficult to measure cathepsin activities at neutral pH with the fluorogenic peptides commonly used as substrates in these assays. However, it also offers the chance to develop pH‐selective inhibitors as it has been recently demonstrated for CTSB [28]. Furthermore, it is known that interactions of cathepsins with other proteins or glycosaminoglycans or DNA affect their stability and function [29, 30, 31, 32]. The complex interactions of cathepsins with many environmental factors makes it difficult to predict the quantity, quality, and duration of proteolytic activity of those lysosomal proteases if they are located in nonacidic cell compartments.

Lysosomal proteases have several ways to escape the endolysosomal compartment. First, about 5% of all cathepsins is secreted via the regular secretory pathway [14]. These are proenzymes with only low residual enzyme activity [33]. Either the interaction with stabilizing and activating co‐factors, as suggested for glycosaminoglycans [30], or the activation in micromilieus with low pH might increase their enzyme activity [34]. Second, exocytosis of mature lysosomes leads to secretion of active cathepsins [35, 36, 37]. Bone resorption by osteoclast cathepsin K [38] and the liberation of thyroid hormones from thryoglobulin by cathepsins B and K [39] are examples for intensive extracellular cathepsin activity. Besides these well‐established events of extracellular cathepsin activity, roles for cathepsins in the cytosol or even in the nucleus have been frequently reported. This topic will be discussed in the remaining sections of this review.

## A brief account of cytosolic cathepsins in dying cells

In the field, it is well agreed upon that the occurrence of cathepsins in the cytosol can cause cell death. According to a concept we would call the classical ‘threshold model’, cell death occurs when the cathepsin concentration exceeds the concentration of their scavenging protease inhibitors (Fig. 1A). This concept is prominently supported by the fact that deficiency of the cytosolic inhibitor of cysteine cathepsins cystatin B (also known as Stefin B) causes death of neurons and thereby a rare childhood form of epilepsy called Unverricht‐Lundborg syndrome [40]. In line with such findings, deficiency of this protease inhibitor in mice resulted in increased cancer cell death in a transgenic breast cancer model [41]. What is very much debated, however, is the mode by which cytosolic cathepsins cause cell death. Proteolytic destruction of anti‐apoptotic proteins, such as Bcl‐xl, and proteolytic activation of proapoptotic proteins, such as the activating truncation of Bid, have been implied in classical apoptotic cell death [42, 43]. Interestingly, upon primary lysosome damage cathepsins, especially CTSB and CTSL, have been shown to support the assembly of the NLR‐family‐pyrin‐domain‐containing‐3 (NLRP3) inflammasome, leading to caspase 1 activation, interleukin 1β/18 maturation with subsequent inflammation and eventually to pyroptotic cell death [5, 44, 45, 46, 47, 48]. The modes of cathepsin‐dependent cell death have been extensively reviewed [6, 49, 50, 51]. Despite this, we would like to point out that studies addressing the topic usually fail to quantify the cytosolic cathepsin concentration, which is largely due to technological limitations. However, we propose that the quantity of cytosolic cathepsins may very well determine which mode of cell death the cell commits to or if small quantities of cathepsin release might even be compatible with cell survival.

### Functions and substrates of nucleo‐cytosolic cathepsins in living cells

In contrast to the ‘threshold model’ in which appearance of cathepsins in the cytosol or nucleus is a purely pathological process (Fig. 1A), accumulating evidence for important cellular functions of cathepsins located in the cytosol or nucleus of ‘healthy’ dividing cells has been reported. The seminal works in this field were the discovery of nuclear CTSL in murine fibroblasts and later the identification of the transcription factor CDP/Cux as a physiological substrate for nuclear CTSL in mouse epidermis [52, 53]. This concept was subsequently extended to cancer cells. As CDP/Cux cleavage drives G1/S transition of the cell cycle, increased expression of nuclear CTSL has been suggested as a new mechanism of cell transformation [54]. In addition, cleavage of p53BP1 by CTSL contributes to genomic instability in triple negative breast cancer cells [55], and a role for nuclear CTSL was demonstrated for cell cycle progression in a colon cancer cell line [56]. In line with these findings, a poor prognosis for patients suffering from colorectal cancer with high levels of nuclear CTSL has been demonstrated [57]. Recently, a role for a CTSL‐CDP/Cux pathway has been suggested for induction of angiogenesis in gastric cancers [58]. In addition to CTSL, other cathepsins have also been implicated in cell cycle regulation. CTSV, also known as CTSL2, has been shown to be present in the nuclei of thyroid carcinoma cells and suggested that it promotes S‐phase progression [59, 60], while CTSB supports chromosome segregation during mitosis [61]. Nuclear transcription repressor TRPS1 (tricho‐rhino‐phalangeal‐syndrome 1, and the nuclear shuttling chaperone BAT3 (Scythe/BAG6)) have been identified as nuclear targets of aspartic CTSD [62]. Those interactions promote cell cycle progression and tumorigenesis. Interestingly, the expression of a catalytically inactive CTSD showed the same effects as the active enzyme, suggesting a proteolysis‐independent nuclear function of this protease.

Further evidence for the functions of nuclear CTSL was obtained in the context of epigenetic regulation [63]. A reorganization of epigenetic markers on the Y chromosome in CTSL‐deficient mouse fibroblasts points to a role of nuclear CTSL in stabilization of histone modifications. Furthermore, nuclear CTSL has been reported to cleave histone H3 in mouse embryonic stem cells that undergo differentiation [64, 65]. More recent studies identified histone H3 and its proteolytically processed forms as key regulators of cellular senescence, cell differentiation, and cell division [61, 66, 67]. Such effects may also explain earlier works that identified nuclear CTSF as a contributor to hepatic stellate cell activation marker expression [68].

Another group of cytosolic targets of cathepsin proteases are components of the cytoskeleton and their interacting partners, i.e., the intracellular domains of transmembrane receptors. For instance, dynamin has been reported as a cytosolic substrate of CTSL. In mouse kidney podocytes, proteolytic processing of dynamin by CTSL leads to a reorganization of the podocyte cytoskeleton. This causes breakdown of the filtration barrier of the glomerulus in mice with LPS‐induced nephrotic syndrome [69]. Synaptopodin turned out to be another cytosolic substrate for CTSL in the kidney [70]. Interruption of this interaction seems to be a key mechanism of the antiproteinuric effect of cyclosporine A treatment [71]. The aminopeptidase CTSH has been shown to clip talin, thereby regulating prostate cancer cell motility [72], and the carboxypeptidase CTSZ (also known as CTSX) regulates T cell morphology by cleaving the β2 integrin LFA‐1 [73]. Interestingly, it was also found that cytosolic CTSS affects Ca^2+^ handling by the endoplasmic reticulum [74].

In summary, there is strong evidence for cathepsin functions in the cytosol and nucleus of living cells. This evidence has been obtained by several independent laboratories over a period of about 20 years. The suggested functions for cytosolic and nuclear cathepsins impact various physiological and pathological processes, although cancer biology is the prime research area addressed. Most of the known cathepsins have been found in the cytosol and/or nucleus albeit with a certain emphasis on murine CTSL and human CTSV (CTSL2).

### The cathepsin route(s) to cytosol and nucleus

Cytosolic and nuclear localization of cathepsins could be due to their escape from normal trafficking routes into membranous intracellular vesicles or their leakage from these organelles. It should be noted that many of the papers concerning nucleo‐cytosolic cathepsin functions do not address this problem. Here, we will discuss three popular escape routes namely (a) mRNA transcript variants produced by the usage of alternative promotors and/or alternative splicing, (b) the use of downstream in‐frame start codons for translation (leaky scanning), and (c) lysosomal membrane permeabilization, describing a leakage out of the lysosome.

#### Transcript variants

**Fig. 3:**
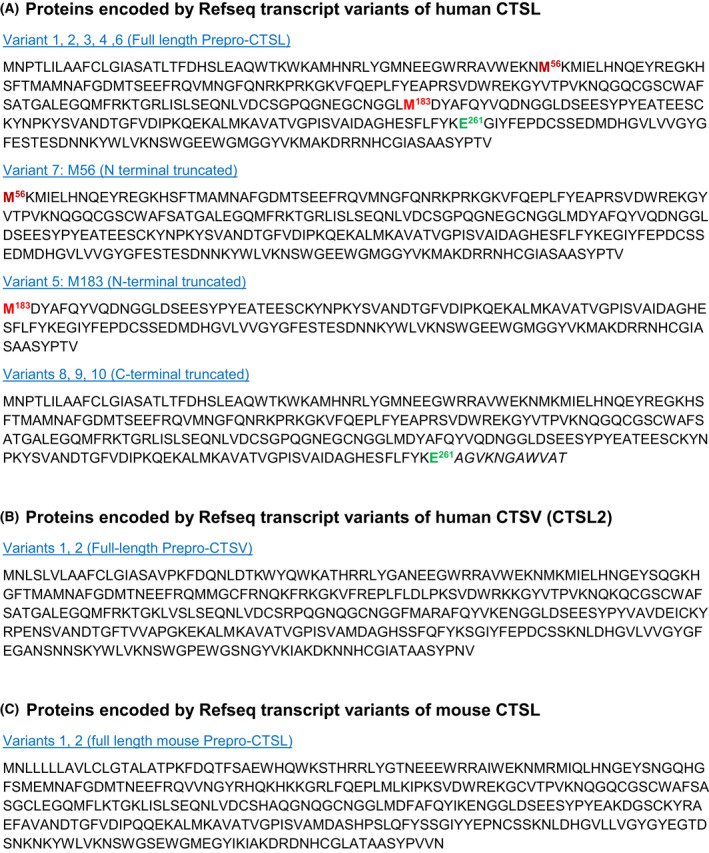
Proteins encoded by the transcript variants of cathepsin L‐like proteases in humans and mice. Due to a gene duplication, the human genome encodes for two cathepsin L enzymes, while the mouse genome encodes for only one. For discussion of this matter, see [14]. Figure presents the Refseq data of the NCBI nucleotide database as of November 2021. (A) Variants for human cathepsin L (CTSL). Note that transcript 7 encodes for an N‐terminal‐truncated variant starting in the CTSL propeptide end encodes the entire single‐chain enzyme. (B) Human cathepsin V (CTSV), also annotated as cathepsin L2 (CTSL2). Note that there are two transcript variants encoding for the identical full‐length enzyme. (C) Mouse cathepsin L (CTSL). Note that there are two transcript variants encoding for the identical full‐length enzyme.

**Fig. 4:**
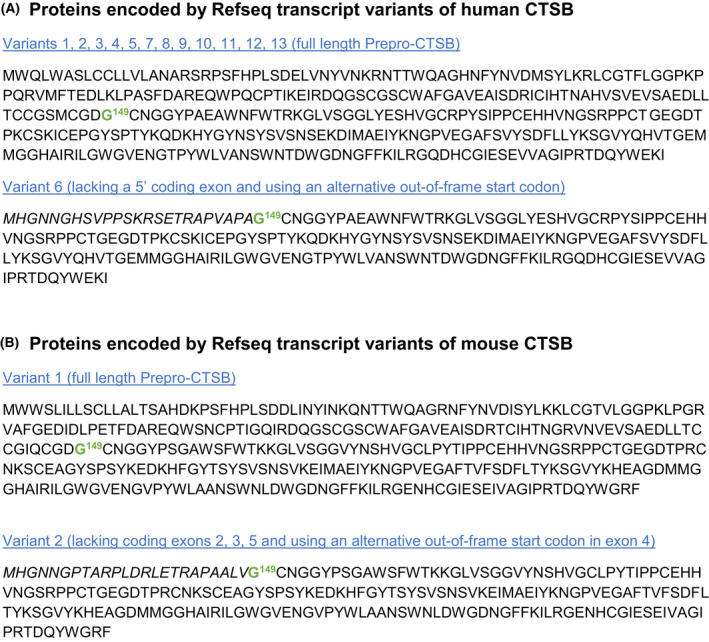
Proteins encoded by the transcript variants of human and mouse cathepsin B (CTSB). Figure presents the Refseq data of the NCBI nucleotide database as of November 2021. (A) Twelve of the 13 human CTSB transcript variants encode for the identical full‐length enzyme. Note that transcript variant 6 encodes an N‐terminal‐truncated protein starting at an out‐of‐frame initiator methionine in exon 4. Exon 5 is spliced out; therefore, the correct CTSB sequence starts at the exon 4/6 junction. (B) Mouse CTSB. Note that transcript variant 2 encodes an N‐terminal‐truncated protein starting of the identical structure as the human variant 6 enzyme. This implies that those CTSB variants are evolutionary conserved. We assume that these are the variants that have been discovered and functionally explored about 20 years ago [82, 83, 84].

Our survey for cathepsin transcript variants in the NCBI nucleotide database as of November 2021 confirmed that cathepsins are synthesized from a variety of transcript variants, e.g., 10 variants of human CTSL and 13 variants for human CTSB (Figs 3 and 4). Unlike other proteins, however, the cathepsin primary structure, i.e., the amino acid sequence, is rarely affected by those mRNA variants. For example, CSTL transcripts, traditionally named A and B, differ in their length because they are controlled by different promotors [75, 76], but both variants lead to translation of the identical protein as the human CTSL open reading frame starts in exon 2. On top of this, alternative splicing produces multiple transcript variants of the 5′untranslated region (5′UTR) of the mRNA. For human transcript variant A, several 5′UTR variants of the first exon are known. They vary in their length of the 5′UTR caused by different splice acceptor sites of exon 1, which are joined to the 5′end of exon 2 [77, 78]. Again, as the initiator methionine of CTSL resides in exon 2, these variants do not affect the amino acid sequence of CTSL. Rather these variants have been reported to differ in their efficiency to be translated into protein. However, contradictory findings have been made about the different translation efficiencies. Some studies observed the highest translation efficiency for the shortest variant [78], whereas others report that the longest variant is favored [79]. Interestingly, it was reported that longest 5′UTR splice variant of human cathepsin L forms an internal ribosome entry site (IRES) structure that may enable favored cap‐independent translation under stress conditions that cause general shutdown of mRNA translation [80]. Indeed, CTSL biosynthesis is maintained under severe cell stress [81]. Interestingly, CTSL transcript variants 8, 9, and 10 predict a C‐terminal truncated protein (Fig. 3A). The significance of this truncation is not explored. More important for our discussion, CTSL variants 5 and 7 encode for N‐terminal‐truncated proteins that would be devoid of the signal peptide needed for ER import of the protein (Fig. 3A). Hence, these transcripts could produce proteases that remain in the cytosol and could be transferred into the nucleus. Yet, the work on nuclear CTSL described in the previous section has been focusing on mouse CTSL and human CTSV, a close relative of CTSL that is also known as CTSL2. Yet, the known transcript variants of CTSV and mouse CTSL all encode for full‐length preproenzymes with ER‐import sequences present (Fig. 3B,C). However, a recent study comparing human CTSL and CTSV excluded the presence of nuclear CTSL but confirmed the presence of nuclear CTSV [60]. Therefore, the significance of the N‐terminal‐truncated CTSL transcript variants 5 and 7 remains enigmatic.

Also, the human CTSB mRNA variants mostly differ at the 5′ UTR as well as the 3′ UTR. Interestingly, CTSB transcript 6 lacks some 5′ coding exons and uses an alternative start codon likely to produce an N‐terminal‐truncated CTSB (Fig. 4). Yet, the functional significance of this variant is unexplored. However, early work on CTSB transcripts identified two variants that lack exon 2 or exon 2/3 that also result in truncated protein specimens [82]. Functional exploration of these variants showed an unexpected mitochondrial localization of truncated CTSB, although minor fractions were also found in cytosol and nucleus [83, 84]. However, in the long‐term expression of those variants was detrimental to cell survival.

For other cathepsins with physiological nucleo‐cytosolic function, as discussed in the previous section, CTSS, CTSZ (CTSX), and CTSD only have mRNA specimens encoding for full‐length proteins. One of the two transcript variants of CTSF would result in an N‐terminal‐truncated protease. Remarkably, six isoforms are listed for CTSH of which 5 would be shortened at the N‐terminus. Yet, NCBI nucleotide database annotates those variants as ‘predicted’. Therefore, experimental validation of their existence is pending. Taken together, there is little experimental evidence that would support transcript variants and/or alternative splicing as a general explanation for the existence of nucleo‐cytosolic cathepsin proteases in living cells.

#### Alternative start codons

**Fig. 5:**
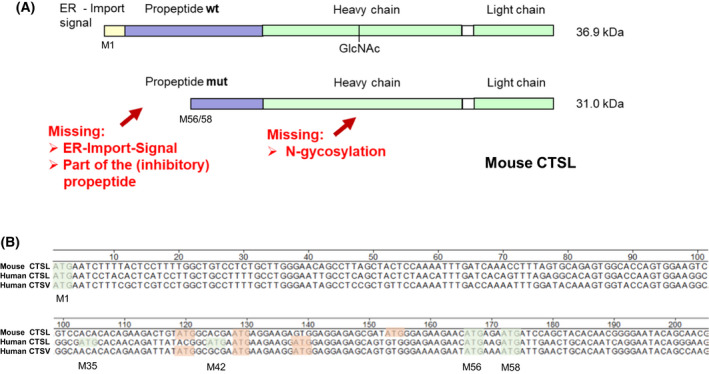
Usage of alternative start codons in cathepsin L‐like proteases in humans and mice. (A) Molecular consequences for initiating the translation of mouse cathepsin L (CTSL) at methionine 56 or 58 (M56/58). (B) Presentation of in‐frame (green) and out‐of‐frame (red) translation initiation sites in mouse and human CTSL and human CTSV (CTSL2) cDNA. Note, that out‐of‐frame start codons hinder efficient initiation at M56/58 and have only short open reading frames encoding for peptides of about 20 amino acids. Human CTSL, however, has two potential in‐frame start codons at M35 and M42 undisturbed by out‐of‐frame initiation sites.

For murine CTSL and later for human CTSV (CTSL2), the usage of alternative start codons has been proposed to explain observation of CTSL in localizations like the cytosol or the nucleus [53, 54, 56, 60]. In its essence, this model implies that translational initiation does not or not efficiently occur at the first start codon of the protease transcript. Instead, downstream in‐frame start codons are used to produce the first methionine on the protein. Figure 5A depicts the consequences of this molecular event: First, the signal needed for ER import would be missing thereby the ribosomes producing the protease would not associate with the ER and remain in the cytosol. Second, a part of the inhibiting propeptide would be missing. Therefore, the access of substrates to the catalytic center of the cathepsin might be allowed. Finally, as N‐glycosylation only occurs in the ER lumen—the typical N‐glycosylations of cathepsins would be missing after such a leaky scanning event. While experiments addressing those isoforms have been focusing on the truncation, the role of the missing N‐glycosylation problem of activity regulation/enzyme activation due to the partially present proregion has been neglected. For instance, the work elucidating the structural basis for CTSL/histone H3 interaction has been performed by producing and purchasing the human and mouse CTSL zymogens in mammalian cells, respectively [65]. Subsequently, those enzymes were activated by traditional methods yielding active proteases as they would be found in the lysosome. Furthermore, the investigation of truncated mouse CTSL and human CTSV has been mostly by expression plasmids encoding only the truncated sequence. Clearly, when using sufficiently strong promotors, it is absolutely possible to produce truncated cytosolic and nuclear cathepsins with those systems. However, *in vivo,* the initiator methionine is present. Figure 5B shows the DNA sequences for the N‐termini of human CTSL, human CTSV (CTSL2), and mouse CTSL. Evidently, all three proteases have conserved alternative in‐frame start codons positioned as amino acid 56 or 58. However, one should note that all three cathepsin sequences encode for three out‐of‐frame start codons that all would produce short peptides, which are likely to be without function. In a systematic study on mouse CTSL cDNA, we have shown that mutating all three out‐of‐frame start codons in addition to the mutation of the regular initiator codon for methionine 1 was required to produce truncated murine CTSL [85]. Interestingly, human CTSL encodes for two potential alternative translational start sites at codons 35 and 42, which would not be hindered by out‐of‐frame start codons. However, as stated above, there is little evidence for nucleo‐cytosolic human CTSL as compared to CTSV [60]. As an *in vivo* approach, the destruction of the canonical start codon of mouse CTSL by knock‐in targeting resulted in production of the modified transcript by the endogenous murine CTSL promotor in various mouse tissues; however, a truncated CTSL isoform was not detected as protein [85]. Furthermore, the expression of the knock‐in transcript did not affect the classical phenotypes of the CTSL knock‐out mice [14, 86]. Taken together, the alternative use of start codons is unlikely to account for the generation of nucleo‐cytosolic cathepsins *in vivo*.

#### Lysosomal membrane permeabilization

Upon their discovery, lysosomes have been interpreted as membranous organelles sequestering the cellular degradation machinery and the acidic conditions required for its function away from the other cell compartments such as cytosol and nucleus [2]. Soon it was realized that lysosome damage was detrimental for cells [87]. These findings initiated an extensive research on the relation of lysosome damage, lysosomal enzyme release, and the modes of cell death as briefly described above. In recent years, it became clear that lysosome functions largely exceed their classical role as garbage disposal. In fact, lysosomes were discovered to represent cellular hubs decisive for cell fate and function. To do so, the lysosomal compartments integrates intracellular energy and nutrient levels with extracellular signals that are relayed by signal transduction pathways such as the phosphatidylinositol‐3‐kinase/mammalian target of rapamycin complex 1(PI3K/mTORC1) axis [88]. In principle, mTORC1 assembles or disassembles at the outer surface of the lysosome aided by multiple proteins associated with the lysosomal membrane as well as by transmembrane proteins such as amino acid transporters [89, 90, 91, 92]. Additional proteins, such as the small GTPase Rab7, and phosphoinositides of the lysosomal membrane, regulate the positioning of this organelle inside cells, which also affects lysosome function [93]. Last but not least, damaged lysosomal membranes can also be repaired [94]. Together, this demonstrates that the lysosomal membrane is highly regulated by various cellular signals either physiologically or stress‐induced.

For our discussion, it is important to note that it was soon realized that lysosomes do not simply ‘explode’ upon exposure to cell stress. Rather, a changed permeability of the lysosomal membrane allows for the release of smaller molecules such as acidophilic dyes, e.g., acridine orange, that change their fluorescence upon increasing pH. For this more moderate transition in lysosomal membrane integrity, the term ‘lysosomal membrane permeabilization*’* (LMP) was coined about 20 years ago [95]. Already back then, it was evident that LMP is not limited to release of small molecules but allows proteins of the lysosomal lumen, such as cathepsin proteases, to be released into the cytosol.

What causes LMP? It is evident that substances damaging the lysosomal membrane cause this phenomenon. Experimentally, so called lysosomotropic agents such as L‐leucyl‐L‐leucine methyl ester, which needs to be activated by cathepsin C, are widely used to initiate LMP [96, 97]. In various pathological context ‘crystals’ that cannot be digested by the lysosome also induce LMP, cathepsin release, inflammation, and eventually immune cell death [45, 46, 98]. There is also solid evidence that multiple types of intracellular stress, like oxidative, mitochondrial, or endoplasmic reticulum stress, can result in LMP [99]. A mechanistic link between oxidative stress and LMP is described in the ‘calpain‐cathepsin’ hypothesis [100]. There is experimental evidence that 4‐hydroxynonenal (4‐HNE), which is a product of lipid peroxidation, covalently modifies HSP70.1. This HSP is known to be important for lysosomal membrane integrity. The 4‐HNE‐modified HSP70.1 is recognized and cleaved by the cytosolic protease calpain. Subsequent to the HSP70.1 cleavage, the lysosome is destabilized and enables LMP with cathepsin release. The most interesting LMP inducers are probably the actions of several phospho‐ and or lipid‐kinases. One should keep in mind that the earliest observation of nuclear CTSL, to our knowledge, was in Ras‐ and Her2‐transformed mouse fibroblasts [52]. The impact of kinases on LMP might occur directly on the lysosomal membrane and its proteins [101, 102, 103] or indirectly by induction of other processes such as mitophagy with subsequent iron‐mediated LMP [104]. Linking the LMP concept with the intracellular signaling pathways, which control fate and function of organelles and whole cells, has the attractive perspective that LMP could be regulated in its timing and its subcellular localization. Furthermore, lysosomal membrane damage can be repaired to a certain extent, while irreversibly damaged lysosomes are removed by lysophagy [94, 105, 106, 107]. By these processes, LMP, which has been a long time considered an irreversible process, can be terminated and eventually reversed. This could explain why lysosome‐derived nucleo‐cytosolic proteases can exist and function in living and dividing cells. A spatially and temporally restricted release of cathepsins from the endolysosomal compartment could also explain the observation that nuclear cathepsins occur dependently on of the cell cycle phase [60, 61, 108]. A prime example is the positioning of lysosomes proximal to the chromatin metaphase plate and their subsequent localized CTSB release for support of chromosome segregation [61]. Taken together, LMP—possibly regulated LMP—would be compatible with many of the existing data on cathepsin functions in the nucleus and cytosol of living cells. As a whole, these studies put in question the uncompromising ‘threshold model’ and favor a model we would call the ‘LMP‐continuity model’ appreciating the compatibility of low‐level LMP with cell survival (Fig. 1B).

## Conclusions

Here, we reviewed the evidence for the existence and function of lysosomal cathepsin proteases in cellular compartments unfavorable to their activity and function. The main focus of our discussion were the routes by which cathepsins might enter the nucleocytoplasm. Yet, many questions and also contradictory findings remain. The challenge is to quantitatively measure nucleo‐cytosolic cathepsins and their activity in the abundant presence of their lysosomal forms. High‐resolution time‐lapse imaging and advanced cell fractionation methods may lead the way. It might be worthwhile to commit resources to the study of so far unexplored transcript and splice variants as well as the translational regulation of cathepsins. Yet, our best current understanding of the facts implies that LMP in only a few lysosomes, which might even be specifically positioned inside cells, releases lysosomal cathepsins to act transiently in a spatially restricted environment. Within this local environment, cathepsin concentrations may exceed the concentration of their endogenous inhibitors, i.e., the cystatins. This resolves in the idea that—in contrast to the classical ‘threshold model’—LMP occurs in distinct degrees inside the cell (Fig. 1B). In this model, high levels of lysosomal leakage are still causing various types of cell death while minor fractions of lysosomes releasing lysosomal content by LMP contribute to important cellular functions. In analogy to the concept of ‘minority MOMP’ (mini‐MOMP) of sublethal mitochondrial outer membrane permeabilization [109], we would like to suggest the term ‘minority LMP’ (mini‐LMP) for the physiological levels of lysosome leakage. The search is on the molecular mechanisms that regulate the timing and extent of mini‐LMP.

## Conflict of interest

The authors declare no conflict of interest.

## Author contributions

TR and MT conceived the topic of this review, TR and MT searched and analyzed the literature, and TR and MT wrote and revised the paper.
